# Future Perspective of Muons: A Quantum Particle Measuring Quantum Processes

**DOI:** 10.1021/acsomega.6c07690

**Published:** 2026-08-28

**Authors:** Adam Berlie, Sayani Biswas, Alex Louat, Rhea Stewart, John Wilkinson

**Affiliations:** 97008 ISIS Neutron and Muon Source, STFC Rutherford Appleton Laboratory, Harwell Campus, Didcot OX11 0QX, United Kingdom

## Abstract

Although considered a niche technique, muon spectroscopy provides unique and complementary insights into a range of different materials, from hard condensed matter to biological samples and everything in between. The muon has a mass of $\frac{1}{9}m_{p}$ or 207*m*_*e*_, and serves as a local probe of quantum states that can provide insight into the bulk properties of materials. While often interpreted in a classical framework, the muon is itself a quantum particle, and it is increasingly common for researchers to take this into account when thinking about muon spectroscopy experiments. In this perspective, we focus on the power of using this quantum treatment of muon spectroscopy, which is a key future direction for the technique.

## Graphical abstract

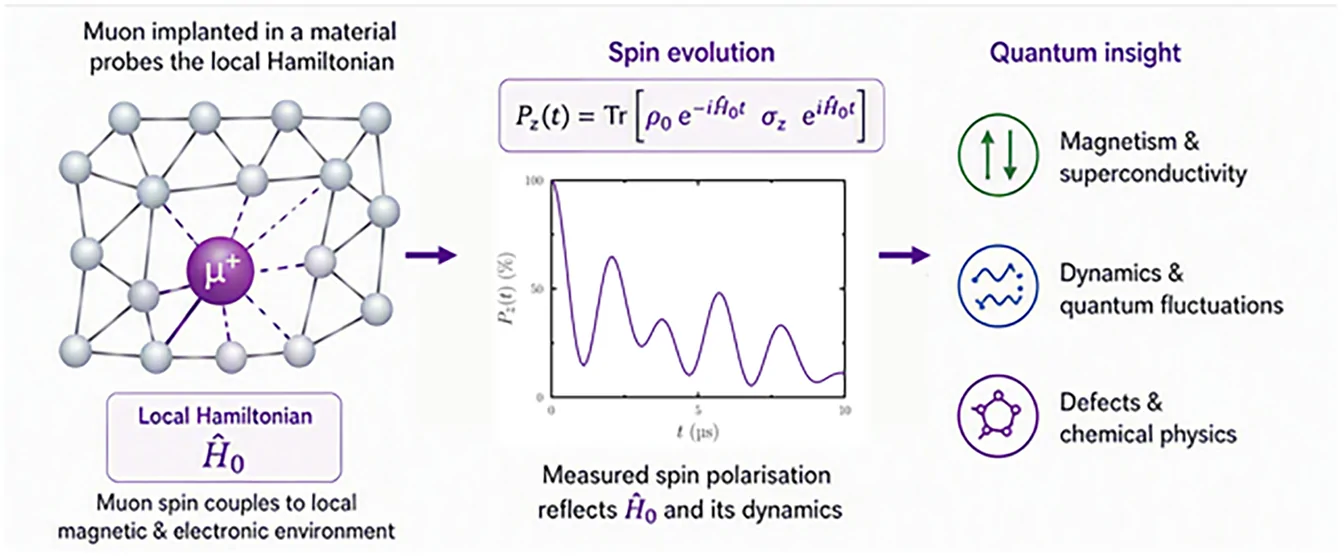

## Introduction

Often at a first glance, the world of muon spin spectroscopy can feel complex and intimidating. For those without a background in physics, the addition of a new particle to our repertoire can feel like a daunting prospect, and bordering closer to the world of particle physics than materials science or chemistry. However, the reality is far from this case: using muons to study materials can provide us with often complementary and in many cases unique information that other experimental techniques either cannot reveal or extract in a complex and indirect way. The hope is that this perspective on the future directions of the muon spin spectroscopy technique provides some food for thought; with readers thinking about how the technique could provide insight into their own research topics as well as to provide a brief amount of information to demystify the use of muons in materials science and chemistry. What we will attempt to convey is the different ways muons can contribute to a variety of problems, but with emphasis on the quantum nature of the muon. It is this quantum behavior that allows us to directly probe quantum processes within systems, where one is essentially measuring the local Hamiltonian of the muon and relating this back to local/bulk physical properties of the system in question.

There is also a growing trend globally for research to be directed toward the solving and tackling of prominent issues or problems and using curiosity driven research to drive economic growth.^1,2^ Many of these key strategic areas hinge on our understanding and discovery of new and novel materials; from understanding quantum systems, to energy storage and optimizing green energy processes, and enhancing our knowledge of chemical systems. In all of these cases, muons have something to contribute, where in general muons tend to focus on blue skies and lower technology readiness levels.

## A Brief Introduction to Muon Spin Spectroscopy

To date, μSR has had an important impact across a number of areas spanning materials physics and chemistry. For example, muons provide an exceptionally powerful probe of static and dynamic magnetism, which has proved essential in understanding bulk and local magnetic ordering, quantum ground states and ionic motion in a wide range of materials such as antiferromagnets, quantum spin liquids and battery cathode materials. The properties of the muon as a probe in both weak transverse and zero magnetic fields has underpinned the development of the technique within superconductors, where muons are uniquely sensitive to the weak magnetic fields associated with unconventional superconductivity and are a key probe of the underlying superconducting pairing mechanism (for a recent review see^3^). The analogy between the muon and a proton (μ^+^ vs. *p* ^+^) or a hydrogen atom (muonium vs. H), as discussed later, has opened the technique up to study defect states and chemical physics; both at a molecular level and in how these species react.

Rather than provide a comprehensive discussion of the history, development and current impact of muon spectroscopy in this persepctive, we refer the reader to a general review article on the technique^4^ and note that further details can be found in several recently published textbooks.^5–7^ Instead, in what follows of this introduction, we will provide a brief overview of some key concepts in muon spin spectroscopy, specifically muon spin relaxation, rotation, and resonance (μSR), to establish context for the discussions that follow.

### Fundamental Properties of Muons

Since the discovery of the muon in 1936, when the famous nuclear physicist, Israel “Isidor” Isaac Rabi famously quipped, “Who ordered that?!”, muons have been a subject of constant fascination. With the first muon spin rotation experiment being performed in 1957,^8^ the groundwork was laid for utilizing the muon as a spectroscopic probe of matter that allows the experimentalist to study the interaction of the muon with its surrounding atomic environment when implanted into chemical samples.

From a particle physics perspective, muons are elementary leptons, but what is key is that muons are spin – $\frac{1}{2}$ unstable particles with a mean lifetime of a few microseconds, that can be implanted into materials to act as a local probe of both nuclear and electronic magnetism. In the presence of a magnetic field, the spin of the muon undergoes Larmor precession, similar to that observed in Nuclear Magnetic Resonance (NMR), but without requiring an equilibrium spin population. The decay product of the muon contains the information that relates back to the behavior of the time evolution of the local nuclear and magnetic fields.

Table 1 summarizes the fundamental properties of muons and how these compare to those of protons and electrons. It is the different gyromagnetic ratios that determine the frequency of the precession and the fields/frequency space that one can measure.

**Table 1:** Physical Properties of Muons, Electrons, and Protons

	Spin	Charge	Mass	Magnetic Moment	Gyromagnetic Ratio, γ/2π (MHz T^–1^)	Mean Lifetime (μs)
Electron	$\frac{1}{2}$	–*e*	*m*_*e*_	647μ_*p*_	28025	∞
Muon	$\frac{1}{2}$	±*e*	207*m_e_* (= 0.11*m*_*p*_)	3.18μ_*p*_	135.53	2.2
Proton	$\frac{1}{2}$	+*e*	1836*m_e_* (= *m*_*p*_)	μ_*p*_	42.576	∞

In a typical NMR experiment, the observable nuclear spin polarization arises from a small thermal population imbalance established in an applied magnetic field and is subsequently manipulated using radio frequency pulses, however muon beams are produced with 100% spin polarization. This means that experiments can be performed in zero applied magnetic fields.

Muons exist in both positive and negative charged forms, denoted by μ^+^ and μ^–^ respectively. When implanted into materials, μ^+^ can be thought of as a light proton and may thermalise to stop in regions of negative electron density, once stopped the muon is then denoted as Mu^+^, whereas μ^–^ behaves similarly to a heavy electron and typically comes to rest close to an atomic nucleus. In both cases, the muon will respond to its local environment, where over many muon lifetimes, one can build up the time evolution of the polarization, relating this back to the behavior of the sample in question. This is the essence of a μSR experiment.

While the majority of muon spectroscopy experiments involve implanting μ^+^ into materials, questions can arise from the motion or diffusion of the light μ^+^, that is held in place by electrostatic or electronic charge. In this case negative muon (μ^–^) experiments are of particular advantage where it is important that the muon is stationary, for example in experiments that aim to measure the diffusion of ions through a battery cathode material.^9^ μ^–^ can also be used to perform negative muon elemental analysis experiments that allow the elemental composition of materials to be determined. This method of elemental mapping offers several advantages over more traditional techniques and is discussed in more detail in the Advancing Knowledge of Chemical Composition section, although the compromise for these two experiments is they often require longer acquisition times.

### Muon States in Matter

Following implantation into a material, positive muons rapidly thermalize through a series of complex inelastic processes into one of several different final states: a bare interstitial muon, a chemically bonded diamagnetic muon (Mu^+^)­or muonium state or paramagnetic muonium (Mu^0^). Muonium (Mu^0^) is a μ^+^–*e* ^–^ bound, neutral, state that can be considered a light isotope of hydrogen. It has a Bohr radius ≈ 1.004*a*_H_, where *a*_H_ is the Bohr radius of hydrogen, and a reduced mass $m_{\mathrm{Mu}}\approx \frac{1}{9}m_{H}$ . Distinguishing between the three states is important and they are illustrated in Figure 1.

**Figure 1:**
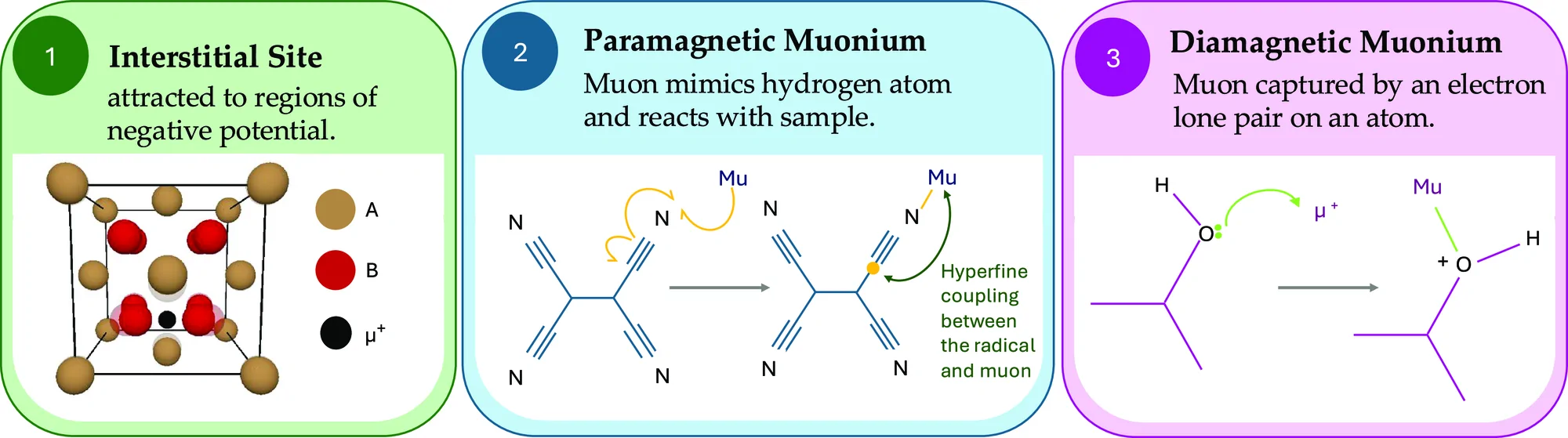
A schematic diagram showing the different muon states that can form in materials 1) a bare muon at an interstitial site; 2) paramagnetic muonium (Mu^0^); and 3) diamagnetic muonium (Mu^+^).

$m_{\mathrm{Mu}}\approx \frac{1}{9}m_{H}$

In the simplest case, the muon remains as a bare positively charged particle occupying an interstitial site within the crystal lattice, where it may induce a local lattice distortion. The muon interacts with nearby nuclear and electronic magnetic moments through dipolar interactions that decay as 1/*r* ^3^ (see section The Muon as a Quantum Probe Embedded in a Quantum System). In molecular systems, the muon may instead form a dative bond with a lone electron pair, analogous to proton binding, or muonium may react with a molecular radical to produce a diamagnetic singlet state. In both cases, the local environment is probed through dipolar and quadrupolar interactions. Alternatively, if paramagnetic muonium forms, it behaves similarly to an isolated hydrogen atom and can participate directly in chemical reactions. A common example is addition across double or triple chemical bonds, producing a paramagnetic radical. There is then a hyperfine interaction between the muon, the radical and surrounding nuclei.

Often one can make a good guess where muons will locate or react from simply looking at the crystal or molecular structure and applying some basic chemical knowledge. However computational techniques offer more precision in calculating the stopping sites.^10^ As a general rule of thumb, in metallic systems most muons remain free, whereas in insulators there is a higher probability of muonium formation.

### Complementarity

It is important to recognize that as we take a more solutions based approach to chemical and physical problems, we understand how to apply the right technique to solve the problem at hand. Because the muon acts as a local magnetic probe while simultaneously sampling the bulk of the material, μSR provides information that is often complementary to other experimental methods.

Like all spectroscopic techniques, μSR is sensitive to a characteristic range of frequencies and time scales. Figure 2a illustrates where μSR lies within the broader landscape of spectroscopic and scattering methods, highlighting the unique frequency window that it occupies and its complementary relationship with other established techniques.

**Figure 2:**
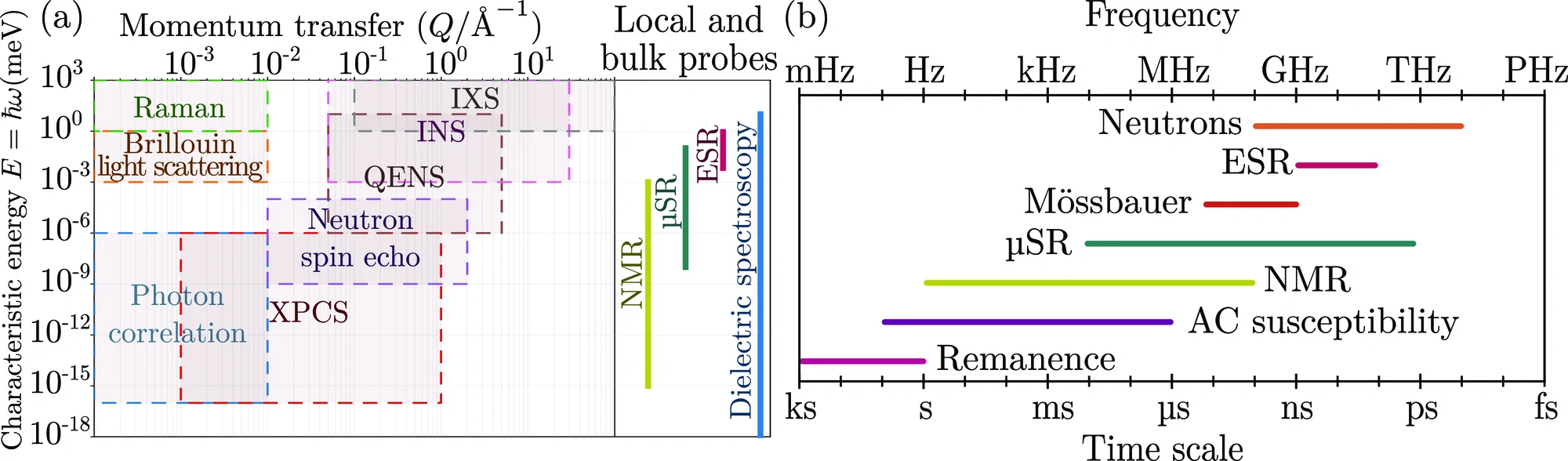
Comparison of the dynamical ranges accessed by selected spectroscopic and scattering techniques. (a) Approximate characteristic energy scales, *E* = *ℏω*, and, for scattering techniques, momentum transfer ranges. Local and bulk probes are shown on the right using an equivalent energy scale derived from their characteristic frequencies. (b) Approximate frequency and corresponding time-scale windows accessible to remanence measurements, AC susceptibility, nuclear magnetic resonance (NMR), muon spin rotation/relaxation (μSR), Mössbauer spectroscopy, electron spin resonance (ESR), and neutron spectroscopy. The displayed ranges are schematic and depend on the instrument, applied field, sample, and experimental protocol. XPCS denotes X-ray photon correlation spectroscopy, QENS quasielastic neutron scattering, INS inelastic neutron scattering, and IXS inelastic X-ray scattering.

Among resonance techniques, perhaps the greatest parallels can be drawn between NMR and Electron Spin Resonance (ESR), where much of the underlying theory between the three techniques is extremely similar. NMR and ESR rely on the application of an external magnetic field to lift the degeneracy of nuclear or electronic spin states through Zeeman splitting, after which radio frequency or microwave excitation perturbs the spin populations away from equilibrium. The subsequent relaxation back to equilibrium is characterized through the spin–lattice (*T*_1_) and spin–spin (*T*_2_) relaxation times. In both cases the spin probes are the nuclei or electrons, respectively.

Like NMR, μSR is a local probe: each implanted muon responds to the magnetic and electronic environment at its stopping site, while the ensemble signal samples the bulk of the material. Unlike neutron or X-ray scattering, μSR does not directly map the response as a function of momentum transfer. The relationship between the local response and reciprocal-space correlations is outlined in the Supporting Information.

This local selectivity is often advantageous. Because the muon occupies well-defined stopping sites, it can selectively probe the dominant phase while remaining relatively insensitive to minor impurity phases. If the stopping sites are understood, the muon effectively acts as an isotopic label for specific regions of the structure.

Figure 2b shows where the muon technique lies in terms of the time scales that other common techniques measure. Of particular note is that the frequencies (or time scales) that μSR probes sit within a gap between AC susceptibility, NMR and neutron scattering. This allows for the MHz frequency range to be probed and behaviors can be traced through a range of different time scales—something that is extremely valuable when considering systems whose dynamics dominate the behavior of interest.

## The Muon as a Quantum Probe Embedded in a Quantum System

It is often convenient to introduce μSR in simple classical language: the muon is implanted into a material, experiences a local magnetic field, and its spin precesses or relaxes in response to that field (similar to NMR and ESR, where the measured field is the vector sum of the applied field and that intrinsic to the sample). Indeed this classical picture is exactly how the technique was initially understood, for example using the result of Kubo and Toyabe’s 1966 thought experiment^11^ to analyze muons implanted in copper.^12^ In 1983 Celio and Meier provided a new interpretation:^13^ they realized that the muon was actually a quantum probe embedded in a quantum mechanical system, and showed that treating the muon in this way with interactions which are beyond simple Zeeman splitting leads to the possibility of an accurate quantitative analysis of diffusion processes within copper. Therefore, while it is useful (and in many routine cases sufficient) to think of μSR in the classical framework, it is ultimately incomplete: the muon is not simply a passive magnetometer placed inside a sample,^4,6^ but rather a quantum particle embedded within a quantum mechanical environment, and the signal measured in a μSR experiment is generated by the time evolution of this coupled quantum system.

Therefore, the physical information contained in a μSR experiment is ultimately determined by the Hamiltonian that governs the muon and its surroundings. In the simplest case, this may indeed reduce to the Zeeman interaction between the muon spin **S**_μ_ and a local magnetic field **B**_loc_,

1 $$H_{Zeeman}=-\gamma_{\mu }S_{\mu }\cdot B_{loc}$$

so that the measured precession frequency can be interpreted directly in terms of the local field. However, there has been considerable work over the past few decades into utilizing other Hamiltonians to probe quantum mechanical processes inside materials: for example, in 1986 the dipole–dipole interactions was used to probe muon-induced distortions in fluorides,^14^ which was later extended to a range of other materials,^15^ and these results were extended further to model systems to quantify quantum mechanical decoherence.^16^ There are also other Hamiltonians can govern the interactions of the muon, including through hyperfine interactions to the local electronic spin density,^17–19^ through quadrupolar terms indirectly via neighboring nuclei, or through time-dependent interactions associated with spin fluctuations, charge motion, diffusion, or chemical dynamics.^12,20,21^ The F–Mu^+^ example shown in Figure 3 provides a useful illustration of this point:^14,16^ in this case, the muon is coupled to its nearest-neighbor fluorine nucleus via the dipole–dipole interaction. Even for this small spin cluster, the coupled energy levels and the resulting polarization function is determined by the quantum Hamiltonian, and this differs substantially from a classical vector-spin description. Therefore, it is important to note that experiments rarely measure “a field” in isolation, but the consequence of quantum evolution under the local Hamiltonian of the muon–matter system.

**Figure 3:**
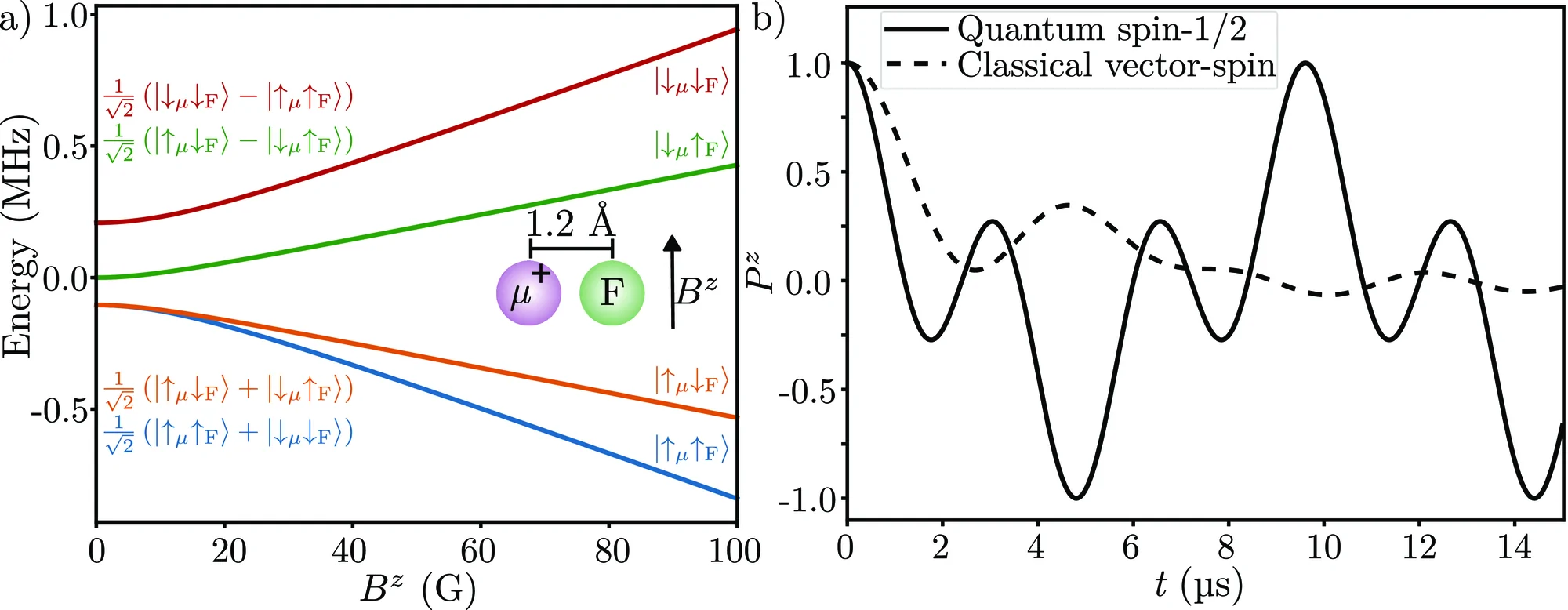
a) Energy levels of the F−μ pair as a function of the magnetic field *B_z_*, with the F–Mu^+^ bond oriented along the *x*-axis. Only the dipolar coupling and an external magnetic field applied along the *z*-axis are included. The inset shows the geometry considered. b) Zero-field (ZF) muon polarization function for unpolarized initial fluorine spins, calculated using quantum spin-1/2 dipolar dynamics and classical vector-spin dipolar dynamics.

In this language, the measured muon polarization is the expectation value of a muon spin operator after time evolution under the Hamiltonian,^6^

2 $$P_{\mu }^{z}\left(t\right)=Tr\left[e^{iH_{0}t}\rho_{0}e^{-iH_{0}t}\sigma_{\mu }^{z}\right]$$

Here ρ_0_ describes the density matrix of the initial state of the muon and its local environment, and $H_{0}$ contains the interactions that entangle, dephase, or relax the muon spin. The experimental asymmetry (which is directly proportional to *P*_μ_(*t*)) is therefore a direct time-domain readout of the quantum interactions governed by $H_{0}$ . This is one of the reasons why μSR is so powerful: the observable is local, microscopic, and dynamical, yet it is measured from an ensemble of muons implanted throughout the bulk of the material.

Thinking in terms of the Hamiltonian also clarifies why different classes of μSR experiment are sensitive to different physical processes and behaviors. Typical Hamiltonians found in μSR are listed in Table 2. For example, in a magnetically ordered material, the dominant term may be the quasi-static coupling of the muon spin to an internal field via the Zeeman Hamiltonian, giving coherent oscillations or a broadened field distribution. In a fluctuating magnet, the important quantity is instead the spectral density of the local field fluctuations at frequencies sampled by the muon spin evolution.^22^ A more technical discussion is provided in the Supporting Information.

**Table 2:** Typical Muon Interaction Hamiltonians

Name	Interaction Hamiltonian	Typical systems
Static Zeeman	$H_{\mathrm{Zeeman}}=-\gamma_{\mu }S_{\mu }\cdot B_{\mu }$	Statically ordered magnets^23,24^ TF-μSR^25,26^
Dynamic Zeeman^a^	$H_{\mathrm{Zeeman}}\left(\tau \right)=-\gamma_{\mu }S_{\mu }\cdot B_{\mu }\left(\tau \right)$	Spin liquids,^27^ frustrated magnets^28^
Dipole–Dipole	$H_{\mathrm{Dip}}=\frac{\mu_{0}}{4\pi }\underset{ij}{\sum }\frac{\hbar \gamma_{i}\gamma_{j}}{r_{ij}^{3}}\left(S_{i}\cdot S_{j}-3\left(S_{i}\cdot {\mathrm{r̂}}_{ij}\right)\left(S_{i}\cdot {\mathrm{r̂}}_{ij}\right)\right)$	Muonic entanglement^14,16,29^ ionic conductors^20^
Quadrupolar	$$H_{Q}=\underset{i}{\sum }\frac{eQ}{\hbar 2I_{i}\left(2I_{i}-1\right)}\underset{\alpha \beta }{\sum }V_{i}^{\alpha \beta }S_{i}^{\alpha }S_{j}^{\alpha }$$	As above, when $I>\frac{1}{2}$ nuclei are involved^16,30,31^
Hyperfine	$H_{\mathrm{HF}}=AS_{\mu }\cdot S_{e}$	Molecular systems^32,33^ Mu^0^ in semiconductors^17,34^

a For dynamic local fields, the relaxation is governed by temporal correlations of the field at the muon site.^22^

In molecular and chemical systems, muonium formation or muonated radical states introduce hyperfine couplings (*A*) that encode information about electronic structure and reactivity. These examples look very different experimentally, but they share the same underlying principle: the physics is determined by the terms that enter the local muonic Hamiltonian.

This perspective is especially important because the muon is part of the system it probes. As discussed in the Muon States in Matter section, the implanted muon may occupy a particular crystallographic site^35–37^ and distort its local environment, form a bond, capture an electron to produce muonium, or act as an analogue of a light hydrogen isotope. These effects are sometimes viewed as complications, however, they are also part of what makes the technique distinctive: the muon can act as a deliberately introduced, infinitely dilute quantum defect whose spin evolution reports on how that defect couples to the surrounding material. Rather than treating the muon perturbation only as something to be removed or corrected for, future developments in μSR should increasingly exploit it as a controlled way of accessing local quantum interactions.

A useful analogy is with other magnetic resonance techniques, where the measured resonance frequencies, relaxation rates, and transition probabilities are interpreted through a spin Hamiltonian. The difference in μSR is that the spin probe is implanted initially highly polarized, which allows one to access local quantum dynamics without the need to create a thermally polarized spin population in the sample. In more advanced forms of the technique, such as RF-μSR or microwave control of muonium, the Hamiltonian is not only observed but actively driven, allowing selected transitions, coherence transfer, or coupled spin states to be manipulated.

The future of μSR therefore lies partly in moving from phenomenological relaxation functions toward Hamiltonian-based interpretation. Standard models such as static or dynamic Kubo–Toyabe functions^11^ remain invaluable, but they often make simplifying assumptions: that the muon samples a Gaussian field distribution, that the surrounding spins are classical and unpolarized, and that the muon does not significantly affect the dynamics being measured. As computational methods, electronic structure calculations, quantum spin simulations, and data analysis tools improve, it becomes increasingly realistic to calculate the muon polarization function from microscopic Hamiltonians.^38–42^ This would allow μSR spectra to be connected more directly to stopping sites, local bonding, nuclear-spin networks, electronic charge density, and dynamical processes.

Viewed in this way, the muon is not merely an unusual experimental probe. It is a quantum particle placed inside a quantum material or chemical environment, and the experiment follows the way in which its spin state evolves under the local Hamiltonian. Interpreting μSR through this framework helps unify the diverse applications of the technique, from magnetism and superconductivity to diffusion, charge order, defects, and chemical reactivity. It also provides a natural route for the field to develop: by treating the muon not as a passive observer of materials, but as an embedded quantum probe whose dynamics reveal the microscopic interactions that govern material behavior.

The above discussion focuses on idealized systems where the interactions are well-defined and the spin of the muon couples to a single nucleus or electron. As one introduces additional degrees of freedom, as is the case for chemical systems, the interaction between the muon and its local environment becomes more complex. However, as summarized below, the muon and muonium remain powerful probes of quantum chemical states. Realizing their full potential will require a more comprehensive understanding of how these probes interact with and report on their local chemical environments.

## Quantum Chemistry: Implanting μ^+^ and Muonium

The electronic structure of muonium closely resembles that of atomic hydrogen, making it a light isotope of hydrogen (Table 3). This unique property enables muonium to act as a hydrogen analogue in chemical systems, while the intrinsic 100% spin polarization of implanted muons makes μSR a powerful technique for probing chemical reactions and local electronic environments. Unlike atomic hydrogen, which is often difficult and expensive to produce and manipulate experimentally, muons can be readily implanted into gases, liquids and solids using a wide range of sample environments. A recently published textbook by Fleming, McKenzie and Percival provides an excellent review of muon interactions with chemical systems.^5^ Consequently, muonium offers a convenient means of introducing a hydrogen-like probe into materials without the experimental challenges associated with generating atomic hydrogen.

**Table 3:** Relative Mass of Muonium to Hydrogen Isotopes

Isotope	Relative Mass
Mu^0^	1/9
^1^H	1
^2^H	2
^3^H	3

Although muonium exhibits chemistry that is broadly analogous to hydrogen, its much lower mass gives rise to important quantum mechanical differences. The reduced mass increases its zero-point energy, leading to greater quantum delocalization and an enhanced probability of tunneling compared with a proton. As a consequence, muonium samples the potential energy surface differently from hydrogen and can access reaction pathways that are more sensitive to nuclear quantum effects. As interest in the quantum mechanical nature of chemical reactions continues to grow,^43^ these differences provide unique opportunities to investigate the role of quantum effects in chemical reactivity. Additionally, it may also provide more understanding of the zero-point motion and tunneling of the muon within a range of materials, where intrinsic motion of the muon can have effects on the processes measured.^39,44^

The chemistry of muonium has been studied for several decades but remains relatively underutilized. Because it behaves as a light isotope of hydrogen, muonium can be implanted directly into materials where it participates in chemical reactions in much the same way as atomic hydrogen. This enables the formation of transient reaction intermediates and final products to be studied in situ. A particularly well-established example is the addition of muonium across carbon–carbon double bonds, leading to the formation of paramagnetic radical species whose electronic structure and dynamics can subsequently be investigated.

The resonance capabilities of μSR provide an additional level of chemical sensitivity. Hyperfine interactions between the unpaired electron of muonium and neighboring nuclei produce characteristic level-crossing resonances, from which hyperfine coupling constants can be determined directly. Furthermore, applying an external magnetic field allows interactions to be probed at specific frequencies according to ν = γ*B*. For diamagnetic muons the relevant gyromagnetic ratio (γ) is that of the muon, γ_μ_, whereas for paramagnetic muonium the much larger electron gyromagnetic ratio, γ_*e*_, dominates the spin dynamics. Electron spin flips therefore induce rapid dephasing of the muon spin, allowing measurements of the muonium spin–lattice relaxation time (*T*_1_) to probe dynamical processes occurring on significantly shorter time scales than are accessible using diamagnetic Mu^+^ states.

More recently, these unique quantum mechanical properties have been exploited to investigate chemically and industrially relevant systems. By combining the hydrogen-like reactivity of muonium with the sensitivity of μSR, it has become possible to identify transient radical intermediates and characterize local electronic environments.^5^ A recent example of this has been to probe complex reaction mechanisms in heterogeneous catalysts.^45^ These capabilities demonstrate the growing role of muon spectroscopy as a complementary tool for understanding chemical reactivity at the atomic scale.

In many of these systems, the magnetic interactions experienced by the muon are large, highly anisotropic, or strongly dependent on the local chemical environment through hyperfine and dipolar couplings. While conventional μSR measurements provide valuable information through passive observation of the spin evolution, even greater insight can be obtained by actively perturbing the spin Hamiltonian. The application of external stimuli, such as oscillating magnetic fields, enables specific spin transitions to be driven and controlled, providing direct access to otherwise inaccessible aspects of the local quantum state and dynamics. The development of these techniques has significantly expanded the experimental parameter space available to μSR and forms the basis of the pulsed methods discussed in the following section.

### Active Manipulation and Pump–Probe Muon Spectroscopy

If the conventional view of μSR is that the muon spin evolves under the Hamiltonian of its local environment, then active spin manipulation represents the next conceptual step: the experimenter deliberately adds a time-dependent term to the Hamiltonian.^46,47,52,53^ Rather than only observing the natural evolution of the implanted muon, one can use radio frequency, microwave, or optical excitation to drive selected transitions, perturb particular degrees of freedom, and watch how the muon responds. In this sense, the muon is not only a quantum probe embedded in a quantum system, but also a handle through which the local quantum system can be manipulated.

This way of thinking is particularly natural when considering small coupled spin clusters. The F–Mu^+^ example in Figure 3 shows that even a minimal muon–nuclear system has a well-defined quantum level structure^14,16,54,55^ and a polarization function that depends sensitively on the spin Hamiltonian. The difference between the quantum spin– $\frac{1}{2}$ calculation and a classical vector-spin description highlights why the language of energy levels, eigenstates, and driven transitions is not merely decorative: as the muon polarization reflects coherent evolution under a local Hamiltonian, applying an oscillatory field at the appropriate frequency opens up the possibility of addressing specific transitions within that Hamiltonian.

The driven Hamiltonian may be written schematically as^53–55^

3 $$H\left(t\right)=H_{0}+H_{drive}\left(t\right)$$

where $H_{0}$ contains the static local interactions of the muon (examples are given in Table 2) and $H_{\mathrm{drive}}\left(t\right)$ represents the applied electromagnetic excitation. The experimental question then changes subtly: instead of only asking how the muon’s spin evolves due to its environment, one asks how the coupled muon–matter system responds when particular transitions or modes are driven. This is the same basic logic that underpins magnetic resonance more generally, but with the distinctive advantage that in μSR the spin-polarized probe is implanted locally and read out directly in the time domain through the muon decay asymmetry.

The range of possible excitations is summarized schematically in Figure 4. Radio-frequency μSR (RF-μSR) occupies the MHz regime, where the relevant energy scales are typically muon Zeeman, nuclear Zeeman, and low-frequency hyperfine or dipolar transitions. In this regime, RF fields can be used to manipulate diamagnetic muons, drive transitions in coupled muon–nuclear spin systems, or decouple selected nuclear moments from the muon.^46,47,52,53,56–58^ This is especially powerful when the loss of muon polarization does not simply represent irreversible relaxation, but instead reflects coherent transfer of polarization into a nearby spin environment. By applying suitable RF pulses, one can distinguish genuine decoherence from reversible dephasing or coherence exchange within the local spin cluster. Thus, RF-μSR turns the muon from a passive reporter of local fields into an active participant in local quantum-state control.

**Figure 4:**
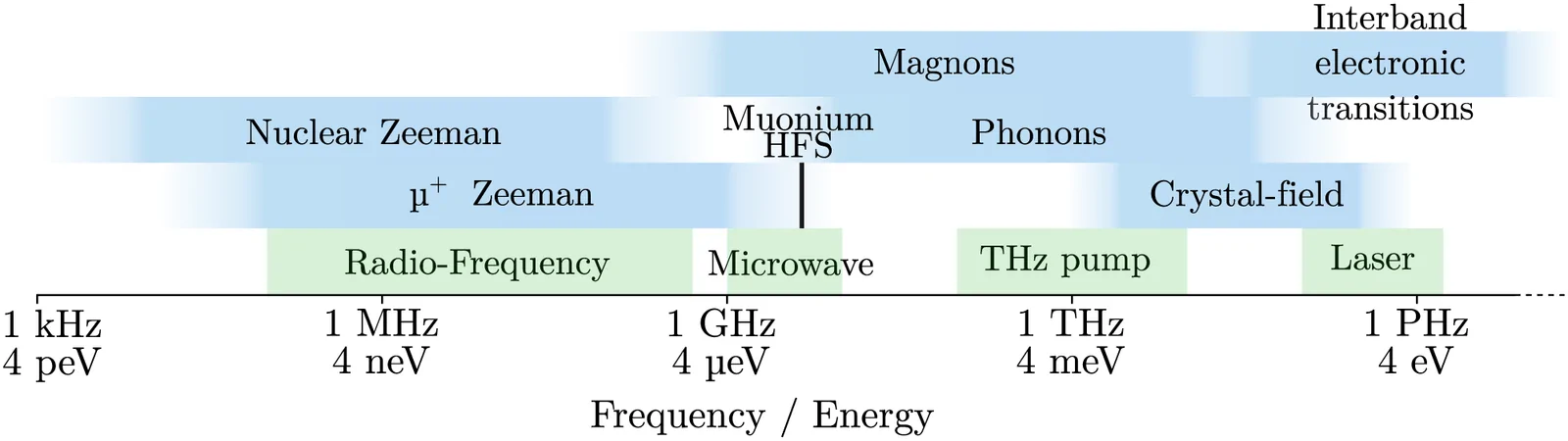
Excitation frequency and energy scales from kHz to PHz, illustrating representative muon–nuclear, muonium hyperfine structure (HFS), collective, and electronic transitions and the corresponding experimental electromagnetic regimes from Radio-Frequency (RF)^46–49^ to optical excitation^34,50,51^ under typical laboratory conditions.

Microwave excitation extends this idea to higher frequencies, particularly for muonium.^48,49,59,60^ Because muonium contains both a muon and an electron, its level structure is governed by the much larger electron gyromagnetic ratio and by the electron–muon hyperfine interaction. The relevant transitions therefore naturally move into the GHz range. Microwave-μSR can access these transitions directly, allowing coherent control of muonium states through Rabi oscillations, Ramsey-type measurements, and related pulse protocols.^60^ In this regime, the muon is part of a hydrogen-like quantum object whose spin and electronic degrees of freedom can be manipulated together. This opens a route to treating muonium not just as a chemical analogue of hydrogen, but as a controllable quantum system embedded in matter.

In contrast to RF and microwave excitation, optical excitation does not primarily drive the muon spin directly. Instead, laser-μSR uses light to perturb the electronic system of the host material,^34,50,51^ for example by generating carriers, changing charge states, or initiating photoinduced chemical processes. The muon simply acts as a local time-domain probe of how those electronic excitations modify the magnetic, hyperfine, or charge-sensitive interactions at its site. This gives laser-μSR the character of a pump–probe experiment: the laser prepares or perturbs the material, and the muon reports on the local microscopic response.

Looking further ahead, THz-pump μSR could probe long-lived changes in the local magnetic, hyperfine, or screening environment produced by collective excitations.^34,61,62^ Where a nonequilibrium excitation population or associated metastable state persists into the nanosecond-to-microsecond regime, its relaxation could be followed as a function of pump–probe delay. This approach would probe the longer-lived response produced by the excitation, rather than the intrinsic picosecond lifetime of the coherently driven THz mode.

The common theme across RF, microwave, optical, and possible THz-pump μSR is the combination of a controlled perturbation with local time-domain readout. RF and microwave fields can act directly on local muon-containing spin states, whereas optical and THz excitation primarily prepare the host material and the muon follows its subsequent relaxation. These approaches therefore extend μSR from equilibrium characterization to time-resolved measurements of how local states and excitation populations evolve after excitation.

## Advancing Knowledge of Chemical Composition

### Experiments with Negative Muons (μ^–^): Explaining Bulk Phenomena with Quantum Processes

Although different to the approach described above with μ^+^, the use of negative muons (μ^–^) has some distinct advantages to providing knowledge on the composition, or changes in composition across a wide range of materials. This can then be used to relate back to the structure–property relationships that researchers are commonly trying to pin down.

Negative muons, unlike positive muons (μ^+^), get captured by the atom of the different elements present inside a given sample, forming a muonic atom. Figure 5 gives a pictorial description of the quantum processes involved during the interaction of a μ^–^ beam with a sample. The muonic atom, thus formed, subsequently emits muonic X-rays, the experimental realization of which has led to the development of a nondestructive elemental analysis technique called muonic X-ray Emission Spectroscopy (μ-XES)[Fn ba-fn1], which is very similar to X-ray Fluorescence (XRF).^66^ This technique, developed back in the 1970s^63,67,68^ has seen a recent resurgence in usage with increasing beam intensities and dedicated experimental setup in large-scale accelerator facilities.^65,69,70^ The key difference between XRF and the μ-XES technique is that the energies of muonic X-rays are much larger than the corresponding electronic X-rays, which makes it possible for muonic X-rays to penetrate from deep inside the sample and reach the detectors, with relatively lower self-absorption inside the sample. For example, the electronic K_α_ X-ray of Cu is at ∼8 keV,^71^ while the **muonic** K_α_ X-ray of Cu is at ∼1514 keV.^65^ Due to this difference, one is able to probe only a few μm inside Cu with XRF, while with muonic X-rays, one can probe as deep as a few millimeters in Cu. In addition, by changing the energy of the μ^–^ beam, one can control the probing depths inside the sample. For example, if we have a sample consisting of a sandwich of Fe and Cu foils with thicknesses 100 and 300 μm respectively, with the Fe layer facing the beam, a μ^–^ beam at 20 MeV/*c* would cause all muons to stop inside Fe layer, leading to emission of muonic X-rays from Fe. However, when a μ^–^ beam at 30 MeV/*c* is bombarded on the same sample, all the muons would stop in the Cu layer giving muonic X-rays of Cu.

Due to the aforementioned properties, the μ-XES technique has been used recently to determine the elemental composition in a nondestructive manner in archeological artifacts.^72–76^ This technique has also been utilized to study battery materials, especially Li-ion batteries (LIBs),^77,78^ the analysis for which is challenging due to the overlapping peaks of muonic K_α_ X-rays of Li (18.7 keV) and muonic L_β_ X-rays of C (18.8 keV). In addition, the detection of light elements, like Li, in thicker materials using this technique has proved to be challenging due to the attenuation/self-absorption of the low-energy muonic X-rays in these thicker samples, as shown in Figure 5a. Also, depending on the setup/muon facility, the detection limit for elements, using this technique is ∼0.5–1 wt %.

After the muonic X-ray cascade process, the muon in the muonic atom undergoes one of two processes: (i) it can be captured by the nucleus which eventually leads to emission of γ rays or (ii) it decays to electrons and hence has a definite lifetime. Very recently, there have been attempts to use an alternative technique using μ^–^ beams, the mean lifetime of μ^–^, to also determine the elemental composition, specifically looking into carbon content in steel samples and ancient Japanese steel swords.^79,80^ These previous publications, claim a detection limit of ∼0.2 wt %, using the muon lifetime technique. The lifetime of μ^–^ is different in different elements; the lifetime decreases with increasing atomic number due to competition with the nuclear capture process.^81–83^ For example, the lifetime of μ^–^ in vacuum is ∼2.2 μs, while that in Cu is ∼160 ns.^82^ An example muon lifetime spectrum has been shown in Figure 5b. The Li muonic X-rays, not visible in Figure 5a are easily identified in Figure 5b. Owing to the challenges in the quantification of Li in LIBs and thicker samples using the muonic X-ray technique (mentioned in the previous paragraph), the usage of the muon lifetime technique to LIBs is being investigated.^84^

**Figure 5:**
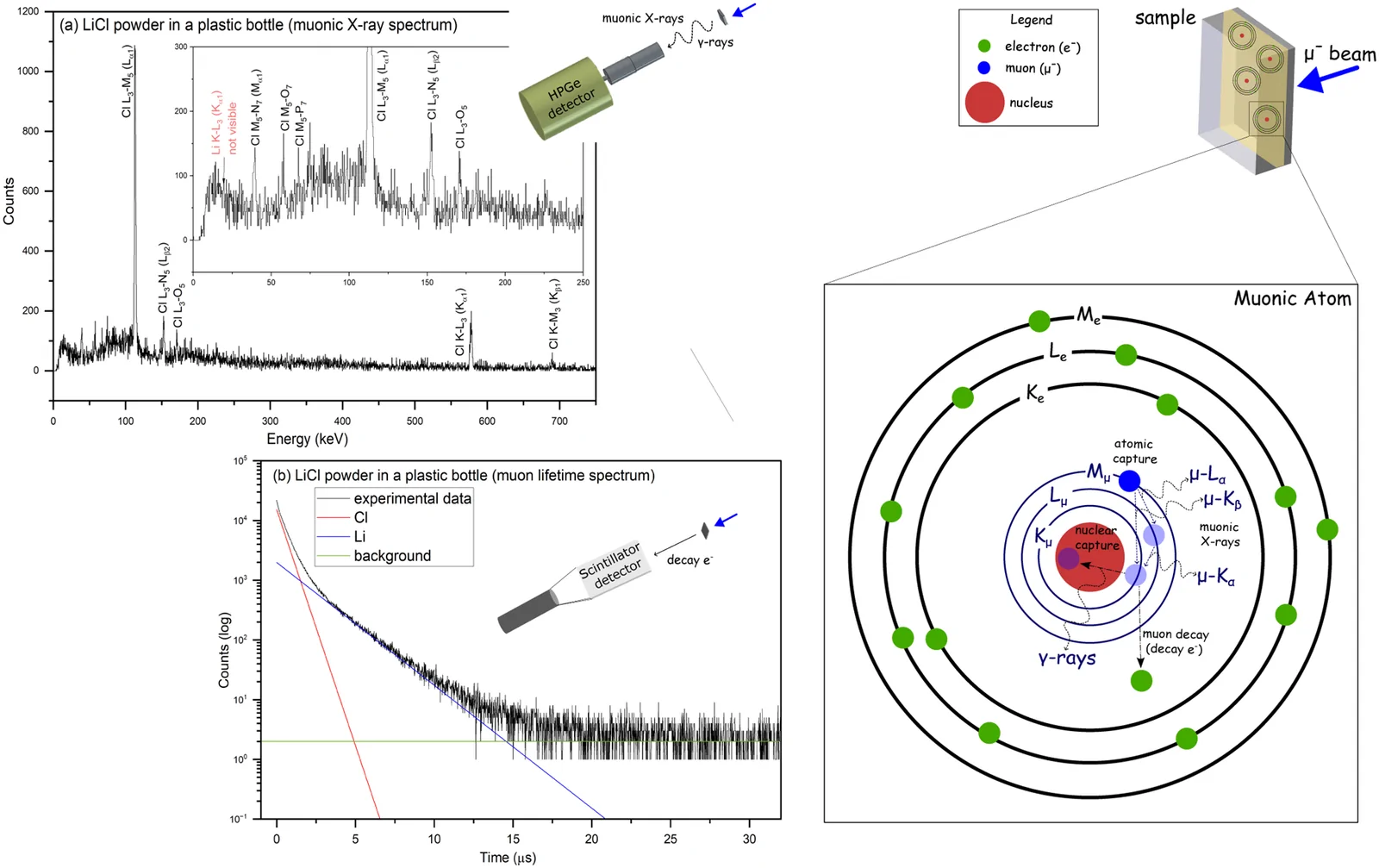
Pictorial representation of the different quantum processes that take place in a μ^–^ experiment once a sample is bombarded with a μ^–^ beam. The electronic and muonic orbitals in the muonic atom have been denoted with subscripts e and μ, respectively. A lithium chloride (LiCl) powder sample, packed in a plastic bottle with dimensions ∼7 × 7 × 12 cm was used to obtain the experimental (a) muonic X-ray spectrum using the MuX spectrometer, an array of high-purity germanium (HPGe) semiconductor detectors and (b) muon lifetime spectrum using the ARGUS spectrometer, an array of plastic scintillator detectors, at the ISIS Neutron and Muon Source. Both the IUPAC (K-L_3_, for example) and Siegbahn (K_α1_, for example) notations, where possible, have been used to denote the characteristic muonic X-ray peaks in (a).

It is the combination of the two aforementioned techniques (more details on these two techniques can be found in the Supporting Information) that allows one to map the elemental distribution across a sample. Many technologically relevant systems rely on the motion or movement of ions, and can comprise a range of different elements spanning from heavy to light. Biological processes, energy storage materials, and some industrial materials tend to focus on the motion/quantification of lighter elements and being able to depth profile heavy and light element composition using the two negative muon techniques allows one to build up a complete picture.

## Final Thoughts

We have shown that muon spin spectroscopy is most useful when we stop thinking of it as a niche technique and start treating it as a genuinely distinctive way of looking at quantum processes within matter. This reframing spans a wide range of materials chemistry and physics; not just ultra low temperature quantum states. The muon does not passively sample a material, instead it is part of the local quantum environment, and its spin evolution gives direct access to the interactions that are important on the scales where a lot of the interesting physics and chemistry sit. That is what makes μSR so valuable: it is not just sensitive, but it is sensitive to the right things, in the somewhat awkward middle ground between local behavior and bulk response where many important materials problems live.

That also helps explain why the technique keeps showing up in such a wide range of areas. Whether the question is about decoherence, diffusion, charge rearrangement, defects, or hydrogen-like chemistry, the common thread is that the important behavior is local, dynamic, and often difficult to get to the bottom of using other methods and techniques. μSR does not replace those techniques; its strength is that it adds something genuinely different and in some cases, special. For most problems, it is precisely the fact that the muon is local and quantum mechanical that makes it useful.

So, the broader point is not that muons need better marketing as a more conventional probe. It is really the opposite. The future success of the field lies in leaning harder into what makes the technique unusual: the quantum nature of the muon, the flexibility of the states it forms in matter, and the fact that it can connect microscopic interactions to real material properties in ways that are hard to do otherwise. If that is where things are heading, then the muon is no longer the oddity in the experimental toolbox; it is becoming one of the more incisive ways of asking what a material is actually doing and how it does it. Rabi may have asked “Who ordered that?”, but at this point it is fair to say the materials community probably should have.

## Supplementary Material



## Footnotes

1 Unfortunately, a common acronym for this technique has still not been agreed upon and is also known by other names, namely, muonic X-ray Analysis (MXA),^63^ Muonic Atom X-ray Spectroscopy (MAXRS),^64^ and Muon Induced X-ray Emission (MIXE).^65^

## Notes

Brief summaries of the different techniques using μ^+^ and μ^–^, together with a technical discussion of the relationship between local μSR relaxation and wavevector-dependent correlations (PDF)

## Notes

In 2001, IUPAC created a document that advised on nomenclature for different states of muons and muonium within matter, we have tried to stick to this. https://www.degruyterbrill.com/document/doi/10.1351/pac200173020377/html
