# Supplementary material for: Future Perspective of Muons: A Quantum Particle Measuring Quantum Processes
Source: ACS Omega. 2026 Aug 28;11(36):53563–73. doi: 10.1021/acsomega.6c07690 (PMC13584516; doi:10.1021/acsomega.6c07690)
Supplement: Supplementary file 1 [file ao6c07690_si_001.pdf]

Supporting Information: Future Perspective of Muons; A Quantum Particle Measuring Quantum Processes

Adam Berlie,* Sayani Biswas, Alex Louat, Rhea Stewart, and John Wilkinson

ISIS Neutron and Muon Source, STFC Rutherford Appleton Laboratory, Harwell Campus, OX11 0QX, United Kingdom

E-mail: adam.berlie@stfc.ac.uk

Muon Spin Relaxation/Resonance/Rotation

μSR is a time-domain probe of local magnetic fields and their fluctuations based on the implantation of 100% spin-polarised positive muons (μ^+) into a material. These muons are typically produced from pion decay at rest ($\pi^+ \rightarrow \mu^+ + \nu_\mu$), which yields a highly polarised beam due to parity violation in the weak interaction. After implantation, the muon loses energy through ionisation and thermalises within picoseconds, coming to rest at an interstitial crystallographic site that depends on the material's electrostatic potential landscape.

The muon's polarisation as a function of time is then governed by Equation 2 in the main text, with the appropriate Hamiltonian (some of which are shown in Table 3 in the main text). The experimental observable in μSR is the time-dependent decay asymmetry $A(t) \propto P_\mu^z(t)$, which tracks the projection of the muon spin polarisation along a chosen quantisation axis z (often chosen to be antiparallel to the beam direction). This is obtained by exploiting the anisotropic decay of the muon: the emitted positron is preferentially emitted along the instantaneous muon

spin direction at the time of decay ($\mu^+ \rightarrow e^+ + \nu_e + \bar{\nu}_\mu$, $\tau \approx 2.197$ μs). Arrays of forward and backward detectors record positron counts as a function of time after implantation, from which $A(t)$ is constructed as a normalised difference signal. The functional form of $A(t)$ contains detailed information about the magnetic field distribution $P(B)$ and its dynamics. For static, randomly oriented local fields (e.g. nuclear dipolar fields), the relaxation is often described by the Kubo–Toyabe function,[1] reflecting a Gaussian field distribution. In contrast, dynamically fluctuating fields produce exponential relaxations that relate directly to the field-field correlation function of the fluctuating field.

Muon spin relaxation measurements are typically performed in zero field (ZF), longitudinal field (LF), or transverse field (TF) geometries to disentangle static and dynamic contributions. In ZF-μSR, the muon's relaxation directly probes internal fields without perturbation, making it highly sensitive to spontaneous magnetic order or slow fluctuations. Application of a longitudinal field can "decouple" the muon spin from static local fields, allowing separation of dynamic relaxation channels and enabling determination of fluctuation spectra. Quantitatively, the relaxation rate often reflects the spectral density of field fluctuations at the muon Larmor frequency, providing access to spin dynamics on timescales ranging from $\sim 10^{-12}$ to 10^{-5} s – an intermediate regime inaccessible to many other probes. TF-μSR allows one to probe the spatial field distribution, and measure the local magnetic susceptibility of materials. Consequently, μSR is widely used to study quantum magnetism, superconducting vortex states (via field distributions in the mixed phase), spin glasses, and critical dynamics near phase transitions, offering site-specific, microscopic insight into magnetic correlations and low-energy excitations.

Local μSR response and wavevector-dependent dynamics

The implanted muon responds to the magnetic field at its stopping site. For a set of surrounding magnetic moments, the fluctuating part of this local field can be expressed in real space as

$$\delta B_\mu^\alpha(t) = \sum_j \sum_\beta A_{\mu j}^{\alpha\beta} \delta S_j^\beta(t) \tag{1}$$

where $A_{\mu j}^{\alpha\beta}$ describes the coupling between component α of the field at the muon and component β of the moment at site j. This coupling may contain dipolar, contact-hyperfine, or other site-dependent contributions.

The same expression can be written in reciprocal space as

$$\delta B_\mu^\alpha(t) = \frac{1}{\sqrt{N}} \sum_{\mathbf{q}} \sum_\beta A_\mu^{\alpha\beta}(\mathbf{q}) \delta S^\beta(\mathbf{q}, t) \tag{2}$$

where N is the number of magnetic sites, $\delta S^\beta(\mathbf{q}, t)$ is the reciprocal-space spin fluctuation, and $A_\mu^{\alpha\beta}(\mathbf{q})$ is the Fourier transform of the real-space coupling, commonly referred to as the muon coupling form factor.

For a longitudinal field applied along z, relaxation of the muon polarisation is driven by fluctuations of the transverse local-field components. Their spectral density may be written as

$$J_\perp(\omega) = \int_{-\infty}^{\infty} \mathrm{d}t \, e^{i\omega t} \left[\left\langle \delta B_\mu^x(t) \delta B_\mu^x(0) \right\rangle + \left\langle \delta B_\mu^y(t) \delta B_\mu^y(0) \right\rangle \right]. \tag{3}$$

In the weak-fluctuation, or Redfield, limit, the longitudinal relaxation rate is proportional to this spectral density at the muon Larmor frequency,

$$\lambda(B_{\mathrm{LF}}) \propto \gamma_\mu^2 J_\perp(\omega_\mu), \qquad \omega_\mu = \gamma_\mu B_{\mathrm{LF}}. \tag{4}$$

Following Keren, the transverse dynamical spin-correlation function may be defined as

$$S_\perp(\mathbf{q},\omega) = \int_{-\infty}^{\infty} \mathrm{d}t\ \cos(\omega t) \left\langle \left\{ \delta S^+_{\mathbf{q}}(t), \delta S^-_{-\mathbf{q}}(0) \right\} \right\rangle , \tag{5}$$

where $\delta S^\pm = \delta S^x \pm i\delta S^y$ and $\{\cdot,\cdot\}$ denotes the anticommutator.[2] Thus, $S_\perp(\mathbf{q},\omega)$ is itself the transverse spin–spin correlation function expressed in wavevector and frequency space. Substituting Eq. 2 into Eq. 3, and using Eq. 4, gives schematically for an approximately isotropic response

$$\lambda(B_{\mathrm{LF}}) \propto \sum_{\mathbf{q}} \left| A^\perp_\mu(\mathbf{q}) \right|^2 S_\perp(\mathbf{q},\omega_\mu). \tag{6}$$

Here, $S_\perp(\mathbf{q},\omega)$ contains the transverse spin correlations, whereas $\left| A^\perp_\mu(\mathbf{q}) \right|^2$ gives the stopping-site-dependent coupling weight. Equation 6 therefore shows that the muon response is generally a coupling-weighted sum over wavevector rather than a measurement at one selected $\mathbf{q}$. The weighting is not necessarily uniform: the crystallographic muon site and the symmetry of the dipolar or hyperfine coupling can enhance or suppress contributions from particular regions of reciprocal space. However, the muon measurement alone does not usually provide a unique momentum-dependent spectrum. Assignment of the relaxation to particular fluctuations therefore generally requires a microscopic model, knowledge of the muon stopping site, or comparison with a momentum-resolved technique such as neutron scattering.[3–5]

Equations 4 and 6 apply when the dynamics can be described by a well-defined longitudinal relaxation rate. For slowly fluctuating, strongly coupled, spatially heterogeneous, or non-Markovian systems, the full muon polarisation function may be non-exponential and must instead be calculated using an appropriate dynamical model.

Elemental Analysis using negative muons (μ^-)

A beam of μ^- with a particular momentum and energy when bombarded on a sample begins to slow down due to the muons interacting with the atomic electrons in the sample. When it

is slowed down to approximately the orbital velocity of an electron in the atom, the μ^- stops (rather, it gets captured in the atom, as described below) inside the sample at a particular depth. Depending on the initial distribution of the momentum of the μ^- beam, the depth at which the μ^- stops also has a distribution. The negative charge of μ^- allows it to be captured by the atom in the sample, forming muonic atom, due to the attractive field of the positively charged nucleus at the centre of the atom. Since the muon is ≈207 times heavier than an electron (see Table 1 in the main text), the muonic orbitals, thus formed, have a much smaller orbital radius ($r_n = \frac{4\pi\epsilon_0 n^2\hbar^2}{me^2}$[6]) than the corresponding electronic orbitals with the same principal quantum number (n). Once the muon is captured in one of the higher muonic orbitals (usually $n_\mu = 14$[7]), it begins to de-excite to its ground state ($n_\mu = 1$) by emitting a series of characteristic muonic X-rays, corresponding to the atom in which it was captured. The experimental apparatus used to detect these muonic X-rays usually consists of an array of High Purity Germanium detectors (HPGe), a semiconductor detector typically used for high-resolution gamma-ray spectroscopy experiments. A publicly available simulation software, called MuDirac,[8] is able to compute the muonic X-ray transition energies for all elements using the Dirac equation, and this has now been integrated in a user-friendly analysis software for analysing muonic X-ray data.[9] These muonic X-rays are characteristic of the elements constituting the sample, however elements with concentrations $\lesssim$0.5-1.0 wt% seems challenging to be detected using this technique (the detection limit varies with the experimental setup and the facility). By analysing the muonic X-ray spectrum from the sample and with the help of calibration standards (with composition similar to the sample under study), one can determine the elemental composition of the sample under study. By varying the momentum/energy of the μ^- beam (done by varying the currents in the various optical elements in the beamline), one can probe different depths inside the sample. This technique, called muonic X-ray Emission Spectroscopy (μ-XES), thus allows the non-destructive and depth-dependent quantification of the elements constituting the sample.

Figure 5(a) in the main text shows the muonic X-ray spectrum using a lithium chloride (LiCl) powdered sample as an example. This figure shows peaks corresponding to muonic Lyman (μ

K-series), Balmer (μ L-series), Paschen (μ M-series) for Cl. The $K_{\alpha1}$ and $K_{\beta1}$ muonic X-rays for Cl are at 578.53 and 691.57 keV, respectively. The equivalent electronic X-rays for Cl are at 2.622 and 2.816 keV respectively. Chlorine has two stable isotopes (^{35}Cl, and ^{37}Cl), with more than 1% abundance,[10] but since the difference between the muonic $K_{\alpha1}$ peaks is only 0.23 keV (μ-$K_{\alpha1}$ for ^{35}Cl and ^{37}Cl are at 578.53 and 578.30 keV, respectively), which is much smaller than typical HPGe detector resolution at these energies, the two isotopes of Cl cannot be distinguished in this spectrum. However, for the heavier isotopes, such as Cu/Pb, the difference between the μ-K_{α} peaks (both $K_{\alpha1}$ and $K_{\alpha2}$) of the different isotopes is larger than the detector resolution and hence the isotopes are easily identifiable from the different peaks constituting the complex μ-K_{α} peak.[11,12] Since the muonic X-rays have a much higher energy than the electronic X-rays, they can penetrate out of a relatively thick sample (depending on the density of sample, this penetration depth can vary from a few centimetres in light elements to a few millimetres in heavier sample), with low self-absorption (depending on the element in which the muon was captured and the density of the matrix), before reaching the HPGe detectors. This technique is sensitive to all elements with atomic number $Z \geq 3$ (Li and beyond). One of the limitations of this technique is that low energy K_{α} muonic X-rays from the lighter elements can get completely attenuated inside the sample if it is very thick. As shown in Figure 5(a), the $K_{\alpha1}$ peak of Li at 18.7 keV was not visible, as the sample was very thick. This aspect doesn't just affect the lighter elements, but also heavier elements where there will be partial self-absorption in very thick samples, due to the higher energy of muonic X-rays. Thus for an accurate and precise determination of elemental composition, with the μ-XES technique, one needs to take into account such self-absorption effects in the different HPGe detectors, which would vary from case to case. The other limitation is the higher detection limit of $\approx$0.5-1.0 wt% that can be achieved with this technique using the current state-of-the-art detector arrays and μ^- beam rates. Yet another limitation is the relatively long dead-time of HPGe detectors, which affects the counting rates in case of pulsed facilities.

Once the μ^- is in the ground-state muonic orbital ($n_\mu = 1$), there are two possibilities: (i) it can either decay into electrons, or (ii) since it is so close to the nucleus, it can also get captured

by the nucleus. The probability of these two processes depends on the element: muons captured in lighter elements have a higher probability of decaying to electrons, while the muons captured in heavier elements have a higher probability of a nuclear capture.[13] These different quantum processes have been pictorially depicted in Fig. 5 in the main text.

In situation (ii), the muon interacts with a proton inside the nucleus converting it to a neutron and a neutrino. For our LiCl example, Li with $Z = 3$ mostly decays by electrons, but Cl with $Z = 17$ will be transmuted to another nucleus with $Z = 16$ (sulphur, S). Since the newly formed nucleus is in a higher excited state, it de-excites to its ground state by emitting gamma rays. From one of the previous publications,[14] it can be seen that the gamma rays from S isotopes are of much higher energy and hence are not visible in Fig. 5(a). But there are several examples of muonic X-ray studies where the gamma peaks are observed in the case of heavier elements.[11,15,16]

The decay electrons produced in situation (i) can also be used to determine the elemental composition in addition to the muonic X-rays, although this area of research is still in its infancy[17]. These electrons can be detected using an experimental setup consisting of plastic scintillator detectors (same as a μSR setup), giving the muon lifetime spectrum. The lifetime of muon decay varies with the element in which it has been captured: this is $\sim$2.2 μs in vacuum, but only $\sim$60 ns in Pb.[13] Figure 5(b) shows a plot of the decay lifetime in the LiCl sample. Li has a lifetime of $\sim$2.2 μs, while Cl has a lifetime of $\sim$0.561 μs, and the two components are clearly visible in the spectrum. So, the signature of Li, which is not evident from Fig. 5(a) is easily identifiable in Fig. 5(b). Thus, the muon lifetime technique, in comparison to the μ-XES technique, does not suffer from self-absorption effects in thick samples, as the decay electrons have significantly higher energies than the muonic X-rays. Also, a slightly lower detection limit of $\sim$0.2 wt% has been achieved with this technique.[17] In addition, the plastic scintillators unlike HPGe detectors, can run at much higher rates which allows one to utilise the maximum beam rates. However, when a sample contains a large number of elements, the lifetime plot can become very convoluted (due to summation of large number of exponential components) and better algorithms would be required for deconvolution purpose. So, more studies need to be done in order to progress on

this technique.

Thus, by utilising the different quantum processes that are involved when a μ^- beam interacts with a sample, one is able to measure the elemental composition as a function of depth for the sample in question.

Where can you do these experiments

Accelerator facilities are required to produce intense beams of spin-polarised muons for experiments, which can be either pulsed or continuous sources. Currently, there are five major muon facilities across the world: ISIS (pulsed) in the United Kingdom; JPARC-MUSE (pulsed); and MuSIC (continuous) in Japan; PSI (continuous) in Switzerland; and TRIUMF (continuous) in Canada. The availability of muons for research is therefore rather limited. However, new facilities are being developed and MELODY (pulsed) at the CSNS in China is currently under construction.

References

(1) Kubo, R.; Toyabe, T. In Magnetic Resonance and Relaxation; Blinc, R., Ed.; North-Holland, 1967; p 810.

(2) Keren, A. Muons as probes of dynamical spin fluctuations: some new aspects. Journal of Physics: Condensed Matter **2004**, 16, S4603–S4618.

(3) McMullen, T.; Zaremba, E. Positive-Muon Spin Depolarization in Solids. Physical Review B **1978**, 18, 3026–3040.

(4) Blundell, S. J., Lancaster, T., Pratt, F. L., de Renzi, R., Eds. Muon Spin Spectroscopy: An Introduction; Oxford University Press, 2022.

(5) Hillier, A. D.; Blundell, S. J.; McKenzie, I.; Umegaki, I.; Shu, L.; Wright, J. A.; Prokscha, T.;

Bert, F.; Shimomura, K.; Berlie, A.; others Muon spin spectroscopy. Nature Reviews Methods Primers **2022**, 2, 4.

(6) Griffiths, D. J. Introduction to Quantum Mechanics; Pearson Education, 2009.

(7) Knecht, A.; Skawran, A.; Vogiatzi, S. M. Study of nuclear properties with muonic atoms. The European Physical Journal Plus **2020**, 135, 777.

(8) Sturniolo, S.; Hillier, A. Mudirac: A Dirac equation solver for elemental analysis with muonic X-rays. X-Ray Spectrometry **2021**, 50, 180–196.

(9) Bjorklund, M. A.; Biswas, S.; Chandrasekaran, C.; Agoro, T.; Basak, S.; Foxley, S.; Lord, J. S.; Butler, K. T.; Hillier, A. D. EVA - A New Data Analysis Software Package for Negative Muon Elemental Analysis. Journal of Physics: Conference Series **2026**, 3222, 012020.

(10) NuDat Chart of Nuclides. `https://www.nndc.bnl.gov/nudat3/`, Accessed: 2026-06-30.

(11) Gerchow, L.; Biswas, S.; Janka, G.; Vigo, C.; Knecht, A.; Vogiatzi, S. M.; Ritjoho, N.; Prokscha, T.; Luetkens, H.; Amato, A. GermanIum array for non-destructive testing (GIANT) setup for muon-induced x-ray emission (MIXE) at the Paul Scherrer Institute. Review of Scientific Instruments **2023**, 94, 045106.

(12) Ninomiya, K.; Kudo, T.; Strasser, P.; Terada, K.; Kawai, Y.; Tampo, M.; Miyake, Y.; Shinohara, A.; Kubo, K. M. Development of non-destructive isotopic analysis methods using muon beams and their application to the analysis of lead. Journal of Radioanalytical and Nuclear Chemistry **2019**, 320, 801–805.

(13) Yamazaki, T.; Nagamine, K.; Nagamiya, S.; Hashimoto, O.; Sugimoto, K.; Nakai, K.; Kobayashi, S. Negative Moun Spin Rotation. Physica Scripta **1975**, 11, 133.

(14) Arole, S.; Armstrong, D. S.; Gorringe, T. P.; Hasinoff, M. D.; Kovash, M. A.; Kuzmin, V.;

Moftah, B. A.; Sedlar, R.; Stocki, T. J.; Tetereva, T. Muon capture on $^{35}\mathrm{Cl}$. Phys. Rev. C **2002**, 66, 065501.

(15) Biswas, S. et al. Characterization of a Continuous Muon Source for the Non-Destructive and Depth-Selective Elemental Composition Analysis by Muon Induced X- and Gamma-rays. Appl. Sci. **2022**, 12.

(16) Hofmann, B. A. et al. An arrowhead made of meteoritic iron from the late Bronze Age settlement of Mörigen, Switzerland and its possible source. Journal of Archaeological Science **2023**, 157, 105827.

(17) Ninomiya, K. et al. Development of a non-destructive depth-selective quantification method for sub-percent carbon contents in steel using negative muon lifetime analysis. Scientific Reports **2024**, 14, 1797.
